# New Health Technologies: A UK Perspective: Comment on "Providing Value to New Health Technology: The Early Contribution of Entrepreneurs, Investors, and Regulatory Agencies"

**DOI:** 10.15171/ijhpm.2017.59

**Published:** 2017-05-09

**Authors:** Nassim Parvizi, Sahar Parvizi

**Affiliations:** ^1^ Oxford University Hospitals NHS Foundation Trust, Oxford, UK.; ^2^ Moorfields Eye Hospital NHS Foundation Trust, London, UK.

**Keywords:** Health Technology, Health Technology Assessment (HTA), National Institute of Health Research (NIHR), National Institute for Health and Care Excellence (NICE)

## Abstract

New health technologies require development and evaluation ahead of being incorporated into the patient care pathway. In light of the recent publication by Lehoux et al who discuss the role of entrepreneurs, investors and regulators in providing value to new health technologies, we summarise the processes involved in making new health technologies available for use in the United Kingdom.

Health technologies can be used in health promotion, monitoring, prevention and treatment of disease. This includes medicines, medical devices, screening, etc. In the United Kingdom, ahead of a new medicine being licensed for use, it has to gain marketing authorisation for safety and efficacy ie, a licence from either the Medicines and Healthcare products Regulatory Agency (MHRA), the UK medicines and medical devices regulatory body, or the European Medicines Agency (EMA) after which the product can be used for the specified indications. Off-label use of medicines may occur when thought to be in the patient’s best interests either when an unlicensed drug is used or a licensed drug is used outside of its summary of product characteristics. A new medical device requires ‘CE’ (Conformité Européene) marking granted by an authorised European representative under the Medical Devices Directive, currently undergoing revision by the European Commission.^1^ This single validation for medical devices grants a European-wide licence. The regulatory authorities ensure a new health technology undergoes a thorough assessment not only as an intervention, but also for its economic value. Decision analytic modelling can provide an indication of the potential cost-effectiveness using current evidence-base, and help to inform further research priority.^2^

The paper ‘Providing Value to New Health Technology: The Early Contribution of Entrepreneurs, Investors, and Regulatory Agencies’ discusses how entrepreneurs of new health technologies compromise to expedite sales and generate revenues, and how investors often support technologies that generate health gains by accident and not by design. Lehoux and colleagues also discuss how health technology assessments (HTAs) have been widely relied upon in decision making by policy-makers once the technology has been released into the market, and so prevents the ability to influence the development of that technology at an earlier stage.^3^

In the United Kingdom, HTAs are supported by the National Institute of Health Research (NIHR). The HTA programme is intended to fund work that would otherwise not be conducted, for example by industry through either commissioned or researcher-led work streams. This includes economic modelling, comparison of multiple different drugs, comparison of drugs with non-drug treatments, assessment of out-of-patent technologies, and testing effectiveness of technologies that are currently used but where the evidence is weak. The extent to which the HTA results are put into practice though vary significantly from study to study. Also, HTAs do not necessarily address the challenges or practicalities of implementation in the publicly funded UK National Health Service (NHS), an aspect funded through other streams such as Health Services and Delivery Research.^4^ All HTA work commissioned and funded by the NIHR is ultimately published in the international journal, *Health Technology Assessment*, which can help inform policy-makers.^5^

For the NHS, the National Institute for Health and Care Excellence (NICE) produce guidance regarding clinical and cost-effectiveness through assessing particular medicines and medical devices when there is geographic variation in the availability of a technology or confusion over its value across the United Kingdom, in particular in England.^6^ One of the many roles of NICE are Technology Appraisals. These are underpinned by Technology Appraisal Reviews (TARs), carried out by centres commissioned through the HTA programme ie, the research is conducted by the HTA followed by recommendations made by NICE, which is unique to the United Kingdom. TARs are whereby NICE committees draw on experts in the field to base their recommendations for guidance on review of available clinical evidence and economic viability ie, how well the technology works in relation to the cost in the NHS helping standardise access to health technologies across the United Kingdom. These look at European and/or UK licensed drugs or approved medical devices. They can be single (single technology for a single indication) or multiple (more than one technology or one technology for more than one indication) which can take approximately 37 or 54 weeks, respectively. This is one of the key ways in which the HTA programme influences NICE guidance. There are four possible recommendation outcomes of a TAR process: recommended, optimised, only in research and not recommended. After the TAR recommendation is made by NICE, the NHS is legally obliged to fund and resource the recommendations within 3 months of its publication or specified date.^7^

We agree with Lehoux et al that policy makers should be involved at an early stage in order to review propositions of new health technologies and expedite innovation policies. NICE have a dedicated process for selecting technologies identified by the NIHR Horizon Scanning Centre at the University of Birmingham whereby they are informed of new drugs in development 20 months ahead of marketing authorisation and 15 months ahead of a new indication.^8^ They also welcome suggestions of new medicinal products (which are to receive a marketing authorisation) through UK PharmaScan or from healthcare professionals, researchers and patients. Their work is based on national and local planning as well as development of commissioning policies for new medicines ie, to help the timetabling of HTAs by NICE, and support local budget planning and formulary development.^9^ If a technology receives formal referral by the Department of Health, NICE can then consider a patient access scheme (financial or outcomes based) and/or flexible pricing proposals (schemes proposed by a company to help facilitate patient access to a technology) in order to be at the forefront of high-end patient care delivery. In the United Kingdom, last year the Department of Health published the Accelerated Access Review, which aims to get a medicine from ‘bench-to-bedside’ for use on England’s NHS quicker, through reducing the process by up to four years.^10^ This would be highly regarded by both entrepreneurs and innovators alike.

There is a lot of uncertainty facing the healthcare system ahead of the UK’s departure from the European Union (EU) where at the time of writing this commentary negotiations are being initiated for the terms and conditions and the financial implications of “Brexit.” In the absence of a UK-EU mutual agreement, there will be a large shortfall in funding for UK Research and Innovation once it is no longer eligible for European Research Council funding.

Health technologies that involve European pathways such as granting of marketing authorisations through the EMA, the Medical Device Directive and the future of medical devices’ ‘CE’ marking will need to be addressed as a matter of urgency to avoid any delays in access to new technologies. It is important for both the UK medical technology industry and NHS to work together to retain influence over future European regulation as one of the largest healthcare providers in the world.^11^ They will also need to be able to continue to negotiate the development of new technologies and prevent any restriction of the ability of UK manufacturers to export medical devices into the large European medical technology market.

Regulatory agencies must maintain their attention towards safety and efficacy, but must be rationalised if they are to afford more efficiency in the face of a rapidly evolving medical technology innovation sector and the flat/reduced NHS hospital budgets available to fund these innovations. This also relies on the development and maintenance of a resilient and adaptable infrastructure so that the healthcare system is not overwhelmed by new technologies and information to monitor their performance is collected accurately and contemporaneously to guide future advances.

## Ethical issues

Not applicable.

## Competing interests

Authors declare that they have no competing interests.

## Authors’ contributions

Both authors contributed equally to the writing of this paper.

## Authors’ affiliations

^1^Oxford University Hospitals NHS Foundation Trust, Oxford, UK. ^2^Moorfields Eye Hospital NHS Foundation Trust, London, UK.
